# CONTEMPLATING TEMPORAL LANDMARKS, SENSING TIME, AND PURSUING A MEANINGFUL LIFE IN ADULTHOOD

**DOI:** 10.1093/geroni/igac059.2901

**Published:** 2022-12-20

**Authors:** DaEun Kim, Hsiao-Wen Liao

**Affiliations:** Georgia Institute of Technology, Atlanta, Georgia, United States; Georgia Institute of Technology, Atlanta, Georgia, United States

## Abstract

Research has shown that experiencing and anticipating temporal landmarks affect one’s goal priorities and motivation. Particularly, milestone birthdays (MBD) remind people of where they stand in life timeline, prompting time experience that has consequences for pursuit of meaning. The current study examines this thesis in the context of adult development and aging. We tested whether (1) thinking about past and future MBDs (vs. a regular birthday) affects young, middle-aged, and older adults’ possession of and search for meaning in life and (2) sense of time, and (3) the association between contemplating MBDs and meaning in life is, at least in part, explained by sense of time. Participants (N = 239) were randomly assigned to one of the three conditions to verbally visualize a past MBD, a future MBD, or an upcoming regular birthday. They then completed a meaning in life questionnaire, which assessed presence of and search for meaning (Steger et al., 2016). Future time perspective (Lang & Carstensen, 1996) and time savoring (Carstensen et al. in prep) were also measured. We found that thinking about past and future MBDs (vs. regular birthdays) prompted people to search for meaning. Thinking about past MBDs (vs. regular birthdays) also elicited a sense that time left in life is limited. Mediation analysis indicated that perceived future time constraints helped explain the relation of recalling past MBDs to search for meaning. Results held when age effects were considered. We draw on motivation theories of temporal landmarks and socioemotional selectivity to discuss the findings.

